# Crystal structure of 4-methyl-2-oxo-2*H*-chromen-7-yl ferrocene­carboxyl­ate

**DOI:** 10.1107/S1600536814022120

**Published:** 2014-10-15

**Authors:** Juan Yu, Lei-Lei Gao, Peng Huang, Dian-Lei Wang

**Affiliations:** a School of Pharmacy, Anhui University of Chinese Medicine, Hefei 230038, People’s Republic of China

**Keywords:** crystal structure, ferrocene, coumarin, pharmacological activity 4-methyl-2-oxo-2*H*-chromene-7-yl ferrocene­carboxyl­ate

## Abstract

The title mol­ecule, [Fe(C_5_H_5_)(C_16_H_11_O_4_)], consists of a ferrocenyl moiety and a 4-methyl­coumarin group linked through an ester unit to one of the cyclo­penta­dienyl (Cp) rings. The two Cp rings are virually parallel, with an angle between the two least-squares planes of 0.74 (16)°. The distances between the Fe^II^ atom and the centroids of the two Cp rings are 1.639 (2) and 1.652 (2) Å. The conformation of the ferrocenyl moiety is slightly away from eclipsed. The dihedral angle between the coumarin ring system and the ferrocenyl ester moiety is 69.17 (19)°. π–π stacking inter­actions involving the benzene rings of neighbouring coumarin moieties, with centroid–centroid distances of 3.739 (2) Å, consolidate the crystal packing.

## Related literature   

For background to ferrocene and its derivatives, see: Štěpnička (2002 ▶). For coumarin and its pharmacological activities, see: Peng *et al.* (2013 ▶). For the crystal structures of related ferrocenyl derivatives, see: Chen & Lu (2004 ▶); Imrie *et al.* (2002 ▶, 2005 ▶).
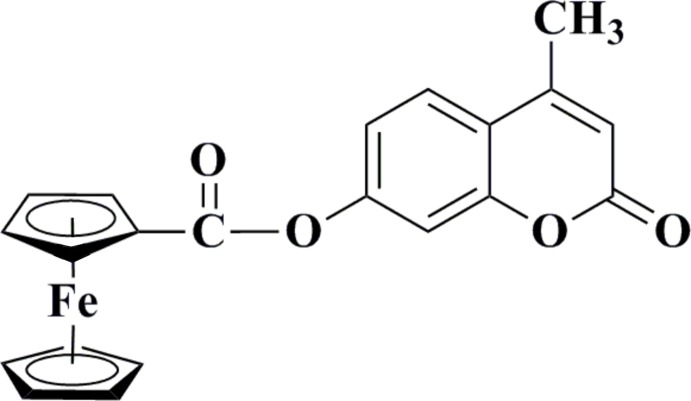


## Experimental   

### Crystal data  

[Fe(C_5_H_5_)(C_16_H_11_O_4_)]
*M* *_r_* = 388.19
Monoclinic, 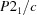

*a* = 7.8678 (11) Å
*b* = 20.294 (4) Å
*c* = 11.1455 (18) Å
β = 108.243 (14)°
*V* = 1690.1 (5) Å^3^
*Z* = 4
Mo *K*α radiation
μ = 0.92 mm^−1^
*T* = 293 K
0.20 × 0.10 × 0.10 mm

### Data collection  

Bruker APEXII CCD diffractometer
Absorption correction: multi-scan (*SADABS*; Bruker, 2007 ▶) *T*_min_ = 0.838, *T*_max_ = 0.914
11857 measured reflections
2979 independent reflections
2398 reflections with *I* > 2σ(*I*)
*R*_int_ = 0.031

### Refinement  

*R*[*F* ^2^ > 2σ(*F* ^2^)] = 0.031
*wR*(*F* ^2^) = 0.087
*S* = 1.16
2979 reflections
236 parameters
H-atom parameters constrained
Δρ_max_ = 0.23 e Å^−3^
Δρ_min_ = −0.18 e Å^−3^

### 

Data collection: *APEX2* (Bruker, 2007 ▶); cell refinement: *SAINT* (Bruker, 2007 ▶); data reduction: *SAINT*; program(s) used to solve structure: *SHELXS97* (Sheldrick, 2008 ▶); program(s) used to refine structure: *SHELXL97* (Sheldrick, 2008 ▶); molecular graphics: *SHELXTL* (Sheldrick, 2008 ▶); software used to prepare material for publication: *SHELXTL*.

## Supplementary Material

Crystal structure: contains datablock(s) I, Global. DOI: 10.1107/S1600536814022120/wm5068sup1.cif

Structure factors: contains datablock(s) I. DOI: 10.1107/S1600536814022120/wm5068Isup2.hkl

**Fig. 1.**
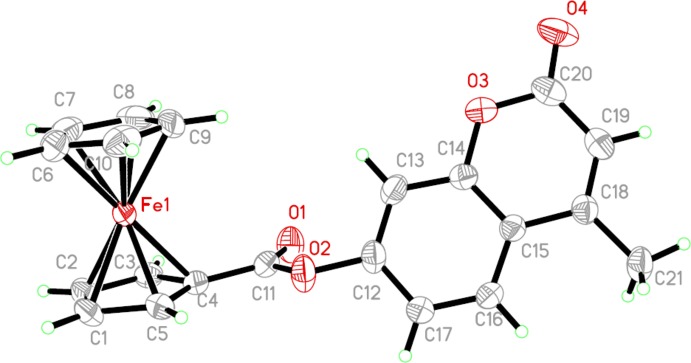
The molecular structure of the title compound showing atoms as ellipsoids at the 30% probability level.

. DOI: 10.1107/S1600536814022120/wm5068fig1.tif
The mol­ecular structure of the title compound showing atoms as ellipsoids at the 30% probability level.

CCDC reference: 1027955

Additional supporting information:  crystallographic information; 3D view; checkCIF report

## supplementary crystallographic information

### S1. Experimental

To a CH_2_Cl_2_ solution (30 ml) of ferrocenylcarboxylic acid (1.0 g, 4.3 mmol), 7-hydroxy-4-methylcoumarin (0.76 g, 4.3 mmol), 1-ethyl-3-(dimethylaminopropyl)carbodimide hydrochloride (EDCI) (0.89 g, 4.5 mmol) and *N*,*N*-dimethylaminopyridine (DMAP) (0.26 g, 2.2 mmol) were added into a 50 ml round bottom flask. Then the reaction mixture was stirred at room temperature. The reaction process was monitored by thin layer chromatography (TLC). After completion of the reaction, the product was extracted with ethyl acetate. The extracts were combined, washed with water and dried over anhydrous Na_2_SO_4_. After removing ethyl acetate under reduced pressure, the crude product was purified by column chromatography using petroleum ether/EtOAc (8:1) as eluent. Yellow crystals were obtained by slow evaporation of an ether/EtOAc (8:1) solution to give 1.03 g (62% yield) of the title compound, ^1^H-NMR (CDCl_3_, δ p.p.m.), 2.48 (s, 2H, CH_3_), 4.34 (s, 5H, C_5_H_5_), 4.57 (s, 2H, C_5_H_2_), 5.00 (s, 2H, C_5_H_2_), 7.18–7.22 (m, 2H, ArH), 7.68 (d, 1H, ArH).

### S2. Refinement

All hydrogen atoms were placed in geometrically idealized positions and constrained to ride on their parent atoms, with C—H = 0.93 Å and *U*_iso_(H) = 1.2 *U*_eq_.

### Crystal data

**Table:**

[Fe(C_5_H_5_)(C_16_H_11_O_4_)]	*F*(000) = 800
*M**_r_* = 388.19	*D*_x_ = 1.526 Mg m^−^^3^
Monoclinic, *P*2_1_/*c*	Mo *K*α radiation, λ = 0.71073 Å
Hall symbol: -P 2ybc	Cell parameters from 3613 reflections
*a* = 7.8678 (11) Å	θ = 2.7–23.4°
*b* = 20.294 (4) Å	µ = 0.92 mm^−^^1^
*c* = 11.1455 (18) Å	*T* = 293 K
β = 108.243 (14)°	Block, yellow
*V* = 1690.1 (5) Å^3^	0.20 × 0.10 × 0.10 mm
*Z* = 4	

### Data collection

**Table:**

Bruker APEXII CCD diffractometer	2979 independent reflections
Radiation source: fine-focus sealed tube	2398 reflections with *I* > 2σ(*I*)
Graphite monochromator	*R*_int_ = 0.031
φ and ω scans	θ_max_ = 25.0°, θ_min_ = 2.0°
Absorption correction: multi-scan (*SADABS*; Bruker, 2007)	*h* = −9→9
*T*_min_ = 0.838, *T*_max_ = 0.914	*k* = −24→24
11857 measured reflections	*l* = −13→12

### Refinement

**Table:**

Refinement on *F*^2^	Primary atom site location: structure-invariant direct methods
Least-squares matrix: full	Secondary atom site location: difference Fourier map
*R*[*F*^2^ > 2σ(*F*^2^)] = 0.031	Hydrogen site location: inferred from neighbouring sites
*wR*(*F*^2^) = 0.087	H-atom parameters constrained
*S* = 1.16	*w* = 1/[σ^2^(*F*_o_^2^) + (0.0432*P*)^2^] where *P* = (*F*_o_^2^ + 2*F*_c_^2^)/3
2979 reflections	(Δ/σ)_max_ = 0.001
236 parameters	Δρ_max_ = 0.23 e Å^−^^3^
0 restraints	Δρ_min_ = −0.18 e Å^−^^3^

### Special details

**Table:**

Geometry. All e.s.d.'s (except the e.s.d. in the dihedral angle between two l.s. planes) are estimated using the full covariance matrix. The cell e.s.d.'s are taken into account individually in the estimation of e.s.d.'s in distances, angles and torsion angles; correlations between e.s.d.'s in cell parameters are only used when they are defined by crystal symmetry. An approximate (isotropic) treatment of cell e.s.d.'s is used for estimating e.s.d.'s involving l.s. planes.
Refinement. Refinement of *F*^2^ against ALL reflections. The weighted *R*-factor *wR* and goodness of fit *S* are based on *F*^2^, conventional *R*-factors *R* are based on *F*, with *F* set to zero for negative *F*^2^. The threshold expression of *F*^2^ > σ(*F*^2^) is used only for calculating *R*-factors(gt) *etc*. and is not relevant to the choice of reflections for refinement. *R*-factors based on *F*^2^ are statistically about twice as large as those based on *F*, and *R*- factors based on ALL data will be even larger.

### Fractional atomic coordinates and isotropic or equivalent isotropic displacement parameters (Å^2^)

**Table:**

	*x*	*y*	*z*	*U*_iso_*/*U*_eq_	
Fe1	0.74731 (4)	0.157566 (14)	0.42513 (3)	0.04477 (13)	
O1	0.4801 (2)	0.07407 (8)	0.61150 (16)	0.0717 (5)	
O2	0.7574 (2)	0.09846 (8)	0.74074 (15)	0.0695 (5)	
O3	0.7829 (2)	−0.13167 (8)	0.81894 (15)	0.0654 (5)	
O4	0.7893 (3)	−0.23952 (9)	0.8434 (2)	0.0910 (6)	
C1	0.7620 (3)	0.25205 (11)	0.4903 (2)	0.0620 (7)	
H1	0.8464	0.2857	0.4811	0.074*	
C2	0.5913 (3)	0.24019 (11)	0.4035 (2)	0.0616 (6)	
H2	0.5372	0.2640	0.3242	0.074*	
C3	0.5129 (3)	0.18719 (11)	0.4494 (2)	0.0549 (6)	
H3	0.3943	0.1683	0.4083	0.066*	
C4	0.6368 (3)	0.16608 (10)	0.5653 (2)	0.0490 (5)	
C5	0.7925 (3)	0.20659 (10)	0.5906 (2)	0.0560 (6)	
H5	0.9001	0.2037	0.6644	0.067*	
C6	0.9160 (4)	0.15047 (13)	0.3194 (3)	0.0734 (8)	
H6	0.9804	0.1869	0.2955	0.088*	
C7	0.7462 (4)	0.12856 (15)	0.2511 (2)	0.0771 (8)	
H7	0.6698	0.1464	0.1707	0.093*	
C8	0.7034 (4)	0.07557 (13)	0.3160 (3)	0.0764 (8)	
H8	0.5928	0.0497	0.2897	0.092*	
C9	0.8462 (4)	0.06495 (12)	0.4251 (3)	0.0758 (9)	
H9	0.8544	0.0310	0.4892	0.091*	
C10	0.9783 (4)	0.11104 (14)	0.4274 (3)	0.0737 (8)	
H10	1.0946	0.1155	0.4929	0.088*	
C11	0.6091 (3)	0.10858 (11)	0.6366 (2)	0.0533 (6)	
C12	0.7538 (3)	0.04467 (11)	0.8189 (2)	0.0573 (6)	
C13	0.7677 (3)	−0.01842 (12)	0.7792 (2)	0.0586 (6)	
H13	0.7736	−0.0268	0.6986	0.070*	
C14	0.7727 (3)	−0.06931 (11)	0.8629 (2)	0.0497 (5)	
C15	0.7663 (3)	−0.05849 (10)	0.9842 (2)	0.0478 (5)	
C16	0.7540 (3)	0.00656 (11)	1.0201 (2)	0.0609 (6)	
H16	0.7509	0.0155	1.1012	0.073*	
C17	0.7463 (3)	0.05785 (12)	0.9382 (2)	0.0633 (7)	
H17	0.7361	0.1010	0.9631	0.076*	
C18	0.7724 (3)	−0.11504 (12)	1.0658 (2)	0.0528 (6)	
C19	0.7797 (3)	−0.17514 (12)	1.0191 (3)	0.0624 (7)	
H19	0.7826	−0.2113	1.0709	0.075*	
C20	0.7833 (3)	−0.18689 (13)	0.8926 (3)	0.0674 (7)	
C21	0.7700 (4)	−0.10441 (13)	1.1988 (2)	0.0731 (8)	
H21A	0.7715	−0.1463	1.2392	0.110*	
H21B	0.6636	−0.0808	1.1972	0.110*	
H21C	0.8733	−0.0794	1.2449	0.110*	

### Atomic displacement parameters (Å^2^)

**Table:**

	*U*^11^	*U*^22^	*U*^33^	*U*^12^	*U*^13^	*U*^23^
Fe1	0.0529 (2)	0.03700 (19)	0.0492 (2)	0.00541 (13)	0.02285 (16)	0.00617 (13)
O1	0.0629 (11)	0.0796 (12)	0.0749 (12)	−0.0128 (10)	0.0250 (10)	0.0156 (10)
O2	0.0814 (12)	0.0671 (11)	0.0539 (10)	−0.0207 (9)	0.0125 (9)	0.0164 (8)
O3	0.0824 (13)	0.0564 (10)	0.0612 (10)	0.0045 (9)	0.0279 (9)	−0.0112 (8)
O4	0.1009 (15)	0.0562 (11)	0.1085 (16)	0.0149 (10)	0.0219 (13)	−0.0211 (11)
C1	0.0792 (19)	0.0351 (11)	0.0761 (18)	0.0034 (11)	0.0306 (16)	0.0030 (12)
C2	0.0711 (17)	0.0467 (13)	0.0711 (17)	0.0193 (12)	0.0282 (14)	0.0145 (12)
C3	0.0530 (14)	0.0545 (13)	0.0617 (15)	0.0134 (11)	0.0244 (12)	0.0002 (12)
C4	0.0602 (15)	0.0440 (12)	0.0487 (13)	0.0060 (10)	0.0253 (12)	−0.0004 (10)
C5	0.0695 (16)	0.0426 (12)	0.0558 (14)	−0.0003 (11)	0.0196 (12)	−0.0036 (11)
C6	0.089 (2)	0.0639 (16)	0.090 (2)	0.0025 (15)	0.0609 (19)	0.0054 (15)
C7	0.100 (2)	0.0832 (19)	0.0534 (16)	0.0245 (17)	0.0307 (16)	−0.0026 (15)
C8	0.082 (2)	0.0568 (16)	0.106 (2)	−0.0087 (14)	0.052 (2)	−0.0307 (16)
C9	0.106 (2)	0.0499 (14)	0.096 (2)	0.0350 (16)	0.067 (2)	0.0242 (14)
C10	0.0569 (16)	0.089 (2)	0.0793 (19)	0.0225 (15)	0.0268 (15)	−0.0039 (16)
C11	0.0627 (16)	0.0550 (14)	0.0498 (14)	0.0015 (12)	0.0285 (13)	0.0003 (11)
C12	0.0632 (16)	0.0573 (14)	0.0481 (14)	−0.0121 (11)	0.0128 (12)	0.0100 (11)
C13	0.0683 (16)	0.0676 (16)	0.0429 (13)	−0.0056 (13)	0.0218 (12)	0.0003 (12)
C14	0.0499 (13)	0.0500 (12)	0.0511 (14)	−0.0007 (10)	0.0187 (11)	−0.0044 (11)
C15	0.0503 (13)	0.0486 (12)	0.0468 (13)	−0.0011 (10)	0.0184 (11)	−0.0001 (10)
C16	0.0886 (18)	0.0535 (14)	0.0466 (14)	−0.0041 (13)	0.0298 (13)	−0.0036 (11)
C17	0.092 (2)	0.0468 (13)	0.0555 (16)	−0.0043 (12)	0.0292 (14)	−0.0028 (11)
C18	0.0517 (14)	0.0540 (14)	0.0550 (14)	0.0031 (10)	0.0199 (12)	0.0082 (11)
C19	0.0591 (16)	0.0518 (14)	0.0778 (18)	0.0053 (12)	0.0234 (14)	0.0106 (13)
C20	0.0589 (16)	0.0546 (15)	0.085 (2)	0.0083 (12)	0.0177 (14)	−0.0042 (15)
C21	0.092 (2)	0.0718 (17)	0.0631 (17)	0.0049 (15)	0.0353 (15)	0.0209 (13)

### Geometric parameters (Å, º)

**Table:**

Fe1—C4	2.019 (2)	C6—C10	1.400 (4)
Fe1—C7	2.024 (2)	C6—H6	0.9793
Fe1—C8	2.026 (2)	C7—C8	1.395 (4)
Fe1—C5	2.027 (2)	C7—H7	0.9791
Fe1—C9	2.034 (2)	C8—C9	1.390 (4)
Fe1—C6	2.037 (3)	C8—H8	0.9793
Fe1—C3	2.037 (2)	C9—C10	1.393 (4)
Fe1—C1	2.041 (2)	C9—H9	0.9794
Fe1—C10	2.042 (2)	C10—H10	0.9796
Fe1—C2	2.047 (2)	C12—C13	1.370 (3)
O1—C11	1.192 (3)	C12—C17	1.376 (3)
O2—C11	1.379 (3)	C13—C14	1.384 (3)
O2—C12	1.402 (3)	C13—H13	0.9300
O3—C14	1.368 (3)	C14—C15	1.387 (3)
O3—C20	1.388 (3)	C15—C16	1.391 (3)
O4—C20	1.208 (3)	C15—C18	1.456 (3)
C1—C2	1.408 (3)	C16—C17	1.373 (3)
C1—C5	1.411 (3)	C16—H16	0.9300
C1—H1	0.9799	C17—H17	0.9300
C2—C3	1.413 (3)	C18—C19	1.335 (3)
C2—H2	0.9799	C18—C21	1.503 (3)
C3—C4	1.419 (3)	C19—C20	1.439 (4)
C3—H3	0.9800	C19—H19	0.9300
C4—C5	1.428 (3)	C21—H21A	0.9600
C4—C11	1.466 (3)	C21—H21B	0.9600
C5—H5	0.9800	C21—H21C	0.9600
C6—C7	1.387 (4)		
			
C4—Fe1—C7	153.19 (12)	C1—C5—Fe1	70.25 (13)
C4—Fe1—C8	120.07 (10)	C4—C5—Fe1	69.05 (13)
C7—Fe1—C8	40.29 (11)	C1—C5—H5	126.3
C4—Fe1—C5	41.35 (9)	C4—C5—H5	126.4
C7—Fe1—C5	164.06 (12)	Fe1—C5—H5	126.4
C8—Fe1—C5	153.89 (12)	C7—C6—C10	107.9 (3)
C4—Fe1—C9	109.67 (9)	C7—C6—Fe1	69.54 (16)
C7—Fe1—C9	67.55 (11)	C10—C6—Fe1	70.10 (15)
C8—Fe1—C9	40.03 (12)	C7—C6—H6	125.7
C5—Fe1—C9	119.71 (11)	C10—C6—H6	126.4
C4—Fe1—C6	165.92 (12)	Fe1—C6—H6	126.2
C7—Fe1—C6	39.94 (11)	C6—C7—C8	108.1 (3)
C8—Fe1—C6	67.30 (11)	C6—C7—Fe1	70.52 (15)
C5—Fe1—C6	126.92 (12)	C8—C7—Fe1	69.91 (15)
C9—Fe1—C6	67.43 (10)	C6—C7—H7	126.4
C4—Fe1—C3	40.94 (9)	C8—C7—H7	125.5
C7—Fe1—C3	118.74 (12)	Fe1—C7—H7	125.9
C8—Fe1—C3	109.32 (11)	C9—C8—C7	108.2 (3)
C5—Fe1—C3	69.04 (10)	C9—C8—Fe1	70.30 (15)
C9—Fe1—C3	129.41 (11)	C7—C8—Fe1	69.80 (15)
C6—Fe1—C3	151.50 (12)	C9—C8—H8	125.3
C4—Fe1—C1	68.54 (9)	C7—C8—H8	126.5
C7—Fe1—C1	126.66 (11)	Fe1—C8—H8	126.0
C8—Fe1—C1	164.88 (13)	C8—C9—C10	107.8 (2)
C5—Fe1—C1	40.59 (9)	C8—C9—Fe1	69.67 (14)
C9—Fe1—C1	152.77 (13)	C10—C9—Fe1	70.30 (14)
C6—Fe1—C1	107.33 (10)	C8—C9—H9	126.7
C3—Fe1—C1	68.16 (10)	C10—C9—H9	125.5
C4—Fe1—C10	128.88 (11)	Fe1—C9—H9	126.0
C7—Fe1—C10	67.28 (12)	C9—C10—C6	108.0 (3)
C8—Fe1—C10	67.12 (12)	C9—C10—Fe1	69.73 (14)
C5—Fe1—C10	108.31 (11)	C6—C10—Fe1	69.76 (15)
C9—Fe1—C10	39.97 (11)	C9—C10—H10	126.3
C6—Fe1—C10	40.14 (11)	C6—C10—H10	125.6
C3—Fe1—C10	167.07 (11)	Fe1—C10—H10	125.9
C1—Fe1—C10	118.65 (11)	O1—C11—O2	122.9 (2)
C4—Fe1—C2	68.50 (9)	O1—C11—C4	126.9 (2)
C7—Fe1—C2	107.62 (11)	O2—C11—C4	110.1 (2)
C8—Fe1—C2	128.22 (13)	C13—C12—C17	121.7 (2)
C5—Fe1—C2	68.47 (10)	C13—C12—O2	120.5 (2)
C9—Fe1—C2	166.40 (13)	C17—C12—O2	117.7 (2)
C6—Fe1—C2	117.71 (10)	C12—C13—C14	117.9 (2)
C3—Fe1—C2	40.49 (9)	C12—C13—H13	121.0
C1—Fe1—C2	40.30 (9)	C14—C13—H13	121.0
C10—Fe1—C2	151.52 (11)	O3—C14—C13	116.2 (2)
C11—O2—C12	117.52 (18)	O3—C14—C15	121.31 (19)
C14—O3—C20	121.64 (19)	C13—C14—C15	122.5 (2)
C2—C1—C5	108.8 (2)	C14—C15—C16	117.2 (2)
C2—C1—Fe1	70.10 (13)	C14—C15—C18	118.70 (19)
C5—C1—Fe1	69.16 (13)	C16—C15—C18	124.1 (2)
C2—C1—H1	125.3	C17—C16—C15	121.4 (2)
C5—C1—H1	125.9	C17—C16—H16	119.3
Fe1—C1—H1	125.6	C15—C16—H16	119.3
C1—C2—C3	108.1 (2)	C16—C17—C12	119.2 (2)
C1—C2—Fe1	69.60 (13)	C16—C17—H17	120.4
C3—C2—Fe1	69.36 (12)	C12—C17—H17	120.4
C1—C2—H2	125.9	C19—C18—C15	118.3 (2)
C3—C2—H2	125.9	C19—C18—C21	122.1 (2)
Fe1—C2—H2	125.8	C15—C18—C21	119.6 (2)
C2—C3—C4	107.8 (2)	C18—C19—C20	123.4 (2)
C2—C3—Fe1	70.14 (13)	C18—C19—H19	118.3
C4—C3—Fe1	68.86 (13)	C20—C19—H19	118.3
C2—C3—H3	126.0	O4—C20—O3	116.0 (3)
C4—C3—H3	126.1	O4—C20—C19	127.3 (3)
Fe1—C3—H3	126.3	O3—C20—C19	116.6 (2)
C3—C4—C5	108.0 (2)	C18—C21—H21A	109.5
C3—C4—C11	123.9 (2)	C18—C21—H21B	109.5
C5—C4—C11	127.9 (2)	H21A—C21—H21B	109.5
C3—C4—Fe1	70.20 (12)	C18—C21—H21C	109.5
C5—C4—Fe1	69.60 (13)	H21A—C21—H21C	109.5
C11—C4—Fe1	121.63 (15)	H21B—C21—H21C	109.5
C1—C5—C4	107.3 (2)		
			
C4—Fe1—C1—C2	−81.64 (15)	C5—Fe1—C6—C10	73.7 (2)
C7—Fe1—C1—C2	72.90 (19)	C9—Fe1—C6—C10	−37.39 (17)
C8—Fe1—C1—C2	45.9 (5)	C3—Fe1—C6—C10	−169.9 (2)
C5—Fe1—C1—C2	−120.3 (2)	C1—Fe1—C6—C10	114.14 (18)
C9—Fe1—C1—C2	−173.15 (19)	C2—Fe1—C6—C10	156.54 (17)
C6—Fe1—C1—C2	112.64 (16)	C10—C6—C7—C8	−0.2 (3)
C3—Fe1—C1—C2	−37.43 (14)	Fe1—C6—C7—C8	−60.10 (18)
C10—Fe1—C1—C2	154.74 (15)	C10—C6—C7—Fe1	59.88 (19)
C4—Fe1—C1—C5	38.64 (14)	C4—Fe1—C7—C6	−170.58 (19)
C7—Fe1—C1—C5	−166.82 (16)	C8—Fe1—C7—C6	−118.6 (2)
C8—Fe1—C1—C5	166.2 (4)	C5—Fe1—C7—C6	39.2 (5)
C9—Fe1—C1—C5	−52.9 (3)	C9—Fe1—C7—C6	−81.18 (18)
C6—Fe1—C1—C5	−127.08 (17)	C3—Fe1—C7—C6	154.99 (15)
C3—Fe1—C1—C5	82.85 (16)	C1—Fe1—C7—C6	71.9 (2)
C10—Fe1—C1—C5	−84.98 (17)	C10—Fe1—C7—C6	−37.73 (16)
C2—Fe1—C1—C5	120.3 (2)	C2—Fe1—C7—C6	112.36 (17)
C5—C1—C2—C3	0.3 (3)	C4—Fe1—C7—C8	−51.9 (3)
Fe1—C1—C2—C3	58.83 (16)	C5—Fe1—C7—C8	157.9 (3)
C5—C1—C2—Fe1	−58.48 (16)	C9—Fe1—C7—C8	37.47 (18)
C4—Fe1—C2—C1	81.76 (15)	C6—Fe1—C7—C8	118.6 (2)
C7—Fe1—C2—C1	−126.44 (17)	C3—Fe1—C7—C8	−86.37 (19)
C8—Fe1—C2—C1	−166.21 (15)	C1—Fe1—C7—C8	−169.44 (17)
C5—Fe1—C2—C1	37.16 (14)	C10—Fe1—C7—C8	80.91 (19)
C9—Fe1—C2—C1	166.6 (4)	C2—Fe1—C7—C8	−128.99 (18)
C6—Fe1—C2—C1	−84.32 (18)	C6—C7—C8—C9	0.4 (3)
C3—Fe1—C2—C1	119.7 (2)	Fe1—C7—C8—C9	−60.04 (18)
C10—Fe1—C2—C1	−51.8 (3)	C6—C7—C8—Fe1	60.48 (18)
C4—Fe1—C2—C3	−37.91 (14)	C4—Fe1—C8—C9	−85.15 (18)
C7—Fe1—C2—C3	113.89 (17)	C7—Fe1—C8—C9	119.1 (2)
C8—Fe1—C2—C3	74.12 (19)	C5—Fe1—C8—C9	−47.3 (3)
C5—Fe1—C2—C3	−82.52 (15)	C6—Fe1—C8—C9	81.44 (18)
C9—Fe1—C2—C3	46.9 (5)	C3—Fe1—C8—C9	−128.94 (16)
C6—Fe1—C2—C3	156.00 (16)	C1—Fe1—C8—C9	153.4 (4)
C1—Fe1—C2—C3	−119.7 (2)	C10—Fe1—C8—C9	37.73 (16)
C10—Fe1—C2—C3	−171.4 (2)	C2—Fe1—C8—C9	−170.38 (15)
C1—C2—C3—C4	−0.2 (3)	C4—Fe1—C8—C7	155.77 (17)
Fe1—C2—C3—C4	58.74 (15)	C5—Fe1—C8—C7	−166.4 (2)
C1—C2—C3—Fe1	−58.98 (16)	C9—Fe1—C8—C7	−119.1 (2)
C4—Fe1—C3—C2	119.3 (2)	C6—Fe1—C8—C7	−37.64 (17)
C7—Fe1—C3—C2	−83.65 (19)	C3—Fe1—C8—C7	111.99 (18)
C8—Fe1—C3—C2	−126.80 (18)	C1—Fe1—C8—C7	34.3 (5)
C5—Fe1—C3—C2	81.00 (16)	C10—Fe1—C8—C7	−81.35 (18)
C9—Fe1—C3—C2	−167.16 (17)	C2—Fe1—C8—C7	70.5 (2)
C6—Fe1—C3—C2	−49.0 (3)	C7—C8—C9—C10	−0.5 (3)
C1—Fe1—C3—C2	37.26 (14)	Fe1—C8—C9—C10	−60.22 (18)
C10—Fe1—C3—C2	161.5 (4)	C7—C8—C9—Fe1	59.72 (18)
C7—Fe1—C3—C4	157.10 (15)	C4—Fe1—C9—C8	113.68 (17)
C8—Fe1—C3—C4	113.95 (16)	C7—Fe1—C9—C8	−37.70 (17)
C5—Fe1—C3—C4	−38.26 (13)	C5—Fe1—C9—C8	158.13 (16)
C9—Fe1—C3—C4	73.59 (19)	C6—Fe1—C9—C8	−81.09 (19)
C6—Fe1—C3—C4	−168.2 (2)	C3—Fe1—C9—C8	71.81 (19)
C1—Fe1—C3—C4	−81.99 (15)	C1—Fe1—C9—C8	−165.2 (2)
C10—Fe1—C3—C4	42.2 (5)	C10—Fe1—C9—C8	−118.6 (2)
C2—Fe1—C3—C4	−119.3 (2)	C2—Fe1—C9—C8	33.9 (5)
C2—C3—C4—C5	0.1 (3)	C4—Fe1—C9—C10	−127.68 (17)
Fe1—C3—C4—C5	59.59 (15)	C7—Fe1—C9—C10	80.94 (18)
C2—C3—C4—C11	−175.0 (2)	C8—Fe1—C9—C10	118.6 (2)
Fe1—C3—C4—C11	−115.4 (2)	C5—Fe1—C9—C10	−83.24 (18)
C2—C3—C4—Fe1	−59.54 (15)	C6—Fe1—C9—C10	37.54 (17)
C7—Fe1—C4—C3	−49.2 (3)	C3—Fe1—C9—C10	−169.56 (16)
C8—Fe1—C4—C3	−85.19 (17)	C1—Fe1—C9—C10	−46.6 (3)
C5—Fe1—C4—C3	118.92 (19)	C2—Fe1—C9—C10	152.6 (4)
C9—Fe1—C4—C3	−128.09 (16)	C8—C9—C10—C6	0.4 (3)
C6—Fe1—C4—C3	156.4 (4)	Fe1—C9—C10—C6	−59.46 (18)
C1—Fe1—C4—C3	80.97 (15)	C8—C9—C10—Fe1	59.82 (17)
C10—Fe1—C4—C3	−168.86 (15)	C7—C6—C10—C9	−0.1 (3)
C2—Fe1—C4—C3	37.51 (14)	Fe1—C6—C10—C9	59.44 (17)
C7—Fe1—C4—C5	−168.1 (2)	C7—C6—C10—Fe1	−59.53 (19)
C8—Fe1—C4—C5	155.88 (16)	C4—Fe1—C10—C9	73.2 (2)
C9—Fe1—C4—C5	112.99 (16)	C7—Fe1—C10—C9	−81.66 (19)
C6—Fe1—C4—C5	37.5 (4)	C8—Fe1—C10—C9	−37.79 (17)
C3—Fe1—C4—C5	−118.92 (19)	C5—Fe1—C10—C9	114.70 (18)
C1—Fe1—C4—C5	−37.95 (14)	C6—Fe1—C10—C9	−119.2 (2)
C10—Fe1—C4—C5	72.22 (18)	C3—Fe1—C10—C9	38.7 (5)
C2—Fe1—C4—C5	−81.41 (15)	C1—Fe1—C10—C9	157.75 (17)
C7—Fe1—C4—C11	69.1 (3)	C2—Fe1—C10—C9	−166.9 (2)
C8—Fe1—C4—C11	33.1 (3)	C4—Fe1—C10—C6	−167.59 (16)
C5—Fe1—C4—C11	−122.8 (3)	C7—Fe1—C10—C6	37.55 (17)
C9—Fe1—C4—C11	−9.8 (2)	C8—Fe1—C10—C6	81.42 (19)
C6—Fe1—C4—C11	−85.3 (4)	C5—Fe1—C10—C6	−126.09 (18)
C3—Fe1—C4—C11	118.3 (3)	C9—Fe1—C10—C6	119.2 (2)
C1—Fe1—C4—C11	−160.7 (2)	C3—Fe1—C10—C6	158.0 (4)
C10—Fe1—C4—C11	−50.6 (3)	C1—Fe1—C10—C6	−83.04 (19)
C2—Fe1—C4—C11	155.8 (2)	C2—Fe1—C10—C6	−47.7 (3)
C2—C1—C5—C4	−0.3 (3)	C12—O2—C11—O1	0.4 (3)
Fe1—C1—C5—C4	−59.37 (15)	C12—O2—C11—C4	−179.32 (19)
C2—C1—C5—Fe1	59.06 (16)	C3—C4—C11—O1	−3.3 (4)
C3—C4—C5—C1	0.2 (3)	C5—C4—C11—O1	−177.3 (2)
C11—C4—C5—C1	174.9 (2)	Fe1—C4—C11—O1	−89.5 (3)
Fe1—C4—C5—C1	60.13 (16)	C3—C4—C11—O2	176.36 (19)
C3—C4—C5—Fe1	−59.97 (15)	C5—C4—C11—O2	2.4 (3)
C11—C4—C5—Fe1	114.8 (2)	Fe1—C4—C11—O2	90.1 (2)
C4—Fe1—C5—C1	−118.4 (2)	C11—O2—C12—C13	72.1 (3)
C7—Fe1—C5—C1	41.8 (4)	C11—O2—C12—C17	−111.6 (3)
C8—Fe1—C5—C1	−171.8 (2)	C17—C12—C13—C14	0.5 (4)
C9—Fe1—C5—C1	155.16 (16)	O2—C12—C13—C14	176.7 (2)
C6—Fe1—C5—C1	72.29 (19)	C20—O3—C14—C13	−178.0 (2)
C3—Fe1—C5—C1	−80.49 (15)	C20—O3—C14—C15	1.4 (3)
C10—Fe1—C5—C1	112.95 (17)	C12—C13—C14—O3	178.7 (2)
C2—Fe1—C5—C1	−36.90 (14)	C12—C13—C14—C15	−0.8 (4)
C7—Fe1—C5—C4	160.2 (4)	O3—C14—C15—C16	−179.2 (2)
C8—Fe1—C5—C4	−53.5 (3)	C13—C14—C15—C16	0.2 (4)
C9—Fe1—C5—C4	−86.45 (17)	O3—C14—C15—C18	0.6 (3)
C6—Fe1—C5—C4	−169.33 (14)	C13—C14—C15—C18	180.0 (2)
C3—Fe1—C5—C4	37.89 (13)	C14—C15—C16—C17	0.8 (4)
C1—Fe1—C5—C4	118.4 (2)	C18—C15—C16—C17	−179.1 (2)
C10—Fe1—C5—C4	−128.67 (15)	C15—C16—C17—C12	−1.1 (4)
C2—Fe1—C5—C4	81.48 (15)	C13—C12—C17—C16	0.4 (4)
C4—Fe1—C6—C7	162.3 (3)	O2—C12—C17—C16	−175.9 (2)
C8—Fe1—C6—C7	37.96 (18)	C14—C15—C18—C19	−1.5 (3)
C5—Fe1—C6—C7	−167.46 (16)	C16—C15—C18—C19	178.3 (2)
C9—Fe1—C6—C7	81.50 (19)	C14—C15—C18—C21	178.6 (2)
C3—Fe1—C6—C7	−51.0 (3)	C16—C15—C18—C21	−1.6 (4)
C1—Fe1—C6—C7	−126.98 (17)	C15—C18—C19—C20	0.5 (4)
C10—Fe1—C6—C7	118.9 (2)	C21—C18—C19—C20	−179.6 (2)
C2—Fe1—C6—C7	−84.58 (19)	C14—O3—C20—O4	178.6 (2)
C4—Fe1—C6—C10	43.5 (5)	C14—O3—C20—C19	−2.4 (3)
C7—Fe1—C6—C10	−118.9 (2)	C18—C19—C20—O4	−179.7 (3)
C8—Fe1—C6—C10	−80.92 (19)	C18—C19—C20—O3	1.4 (4)
