# Supplementary figures and images for: Crystal structure of 4-methyl-2-oxo-2H-chromen-7-yl ferrocene­carboxyl­ate
Source: Acta Crystallogr Sect E Struct Rep Online. 2014 Oct 15;70(Pt 11):m369–70. doi: 10.1107/S1600536814022120 (PMC4257315; doi:10.1107/S1600536814022120)

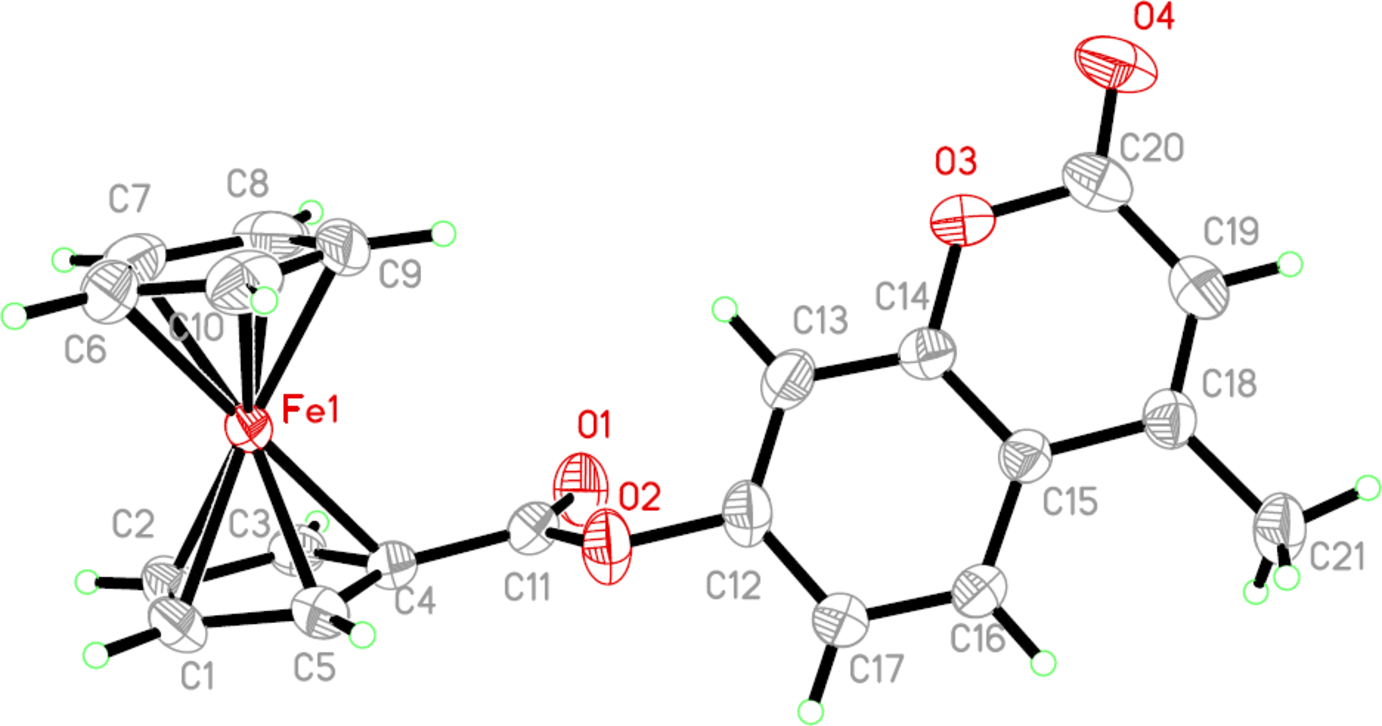

Supplement: Supplementary file 3 [file e-70-0m369-fig1.tif]
